# Evaluation of Two Different Anterior Vitrectomies for Fluid Misdirection Syndrome Secondary to Cataract Surgery Combined with Goniosynechialysis

**DOI:** 10.1155/2020/1934086

**Published:** 2020-03-23

**Authors:** Zhenbin Qian, Yau Kei Chan, Liqing Wei, Bin Zheng, Li Nie, Weihua Pan

**Affiliations:** ^1^Affiliated Eye Hospital, School of Ophthalmology and Optometry, Wenzhou Medical University, Wenzhou, Zhejiang, China; ^2^Department of Ophthalmology, University of Hong Kong, Hong Kong

## Abstract

**Purpose:**

To evaluate two different approaches of anterior vitrectomy combined with hyaloidotomy, zonulectomy, and iridectomy (VHZI) for fluid misdirection syndrome (FMS) secondary to phacoemulsification with intraocular lens implantation combined with goniosynechialysis (phaco-IOL-GSL).

**Methods:**

Nine patients with FMS who developed after a phaco-IOL-GSL procedure, were retrospectively studied from May 2015 to February 2018. They were treated with VHZI, in which 5 cases underwent local anterior vitrectomy via anterior chamber approach, and the others underwent comprehensive vitrectomy via pars plana approach. Main outcome measures were pre- and postoperative intraocular pressure (IOP), number of antiglaucoma medications, and relapse rate.

**Results:**

Incidence of FMS secondary to phaco-IOL-GSL was 1.4% (9 in 652 eyes), which was promptly resolved in all affected cases. VHZI via anterior chamber approach resulted in a significant decrease in the mean IOP from 40.2 ± 9.7 mm Hg at presentation to 15.2 ± 4.8 mm Hg postoperatively (*P*=0.01). The median number of antiglaucoma medications reduced from 3 to 2 (*P*=0.066). Meanwhile, VHZI via pars plana approach also resulted in the reduction of the mean IOP values from 26.0 ± 5.7 mm Hg at presentation to 15.2 ± 3.3 mm Hg postoperatively (*P*=0.092). The median number of antiglaucoma medications also reduced from 3.5 to 1.5 (*P*=0.059). Relapse rate of the treatment through pars plana approach (0%, 0/4) was much lower than that through anterior chamber approach (40%, 2/5), although the difference was not statistically significant (*P*=0.444).

**Conclusions:**

FMS is a rare but severe complication secondary to phaco-IOL-GSL. Compared to VHZI with local anterior vitrectomy via anterior chamber approach, VHZI with comprehensive anterior vitrectomy via pars plana approach might be a more effective procedure to treat FMS. The study has been registered in Contact ClinicalTrials.gov PRS Identifier: NCT04172857.

## 1. Introduction

In the past few years, phacoemulsification with intraocular lens implantation combined with goniosynechialysis (phaco-IOL-GSL) has become significantly popular in the treatment of PACG due to its effectiveness and safety [1–4]. Fluid misdirection syndrome (FMS) is a rare but severe complication observed after phaco-IOL-GSL due to its rapid progression and poor prognosis. To the best of our knowledge, the treatments and outcomes of FMS secondary to phaco-IOL-GSL have not been extensively studied.

The treatment strategies for FMS focused on the re-establishment of the aqueous humor outflow between the anterior chamber and vitreous cavity. Current treatment options for FMS primarily include (1) medications, (2) neodymium:yttrium-aluminum-garnet (Nd:YAG) laser iridotomy with anterior hyaloidotomy in phakic eyes, (3) Nd:YAG laser iridotomy with posterior capsulotomy and hyaloidotomy in pseudophakic eyes, (4) transscleral cyclophotocoagulation, and (5) anterior vitrectomy. However, previous studies showed that the success rate of both medications and laser therapy is low [5–7]. While transscleral cyclophotocoagulation helps to achieve optimum eye resolution in most cases, but it may also damage tissues such as the ciliary processes, sclera, and conjunctiva [8, 9]. As a result, anterior vitrectomy remains as an important choice for FMS. Previous studies suggest that anterior vitrectomy combined with hyaloidotomy, zonulectomy, and iridectomy (VHZI) is effective in creating a permanent passage between the anterior chamber and the vitreous cavity [7, 9–13]. The anterior vitrectomy can be performed via anterior chamber approach or pars plana approach [7, 9–12, 14]. However, a comparative analysis of their efficacy and safety is currently lacking. Here, we investigate and compare the efficacy and safety of VHZI with those two approaches in the eyes of patients suffering from FMS developed after phaco-IOL-GSL.

## 2. Patients and Methods

### 2.1. Patients

The medical records of all patients who developed clinical evidence of FMS after phaco-IOL-GSL, at the Affiliated Eye Hospital of Wenzhou Medical University from May 2015 to February 2018, were reviewed. FMS was diagnosed based on the presence of a central and peripheral shallow anterior chamber with a patent iridotomy and normal or elevated intraocular pressure (IOP). The diagnosis was made only after confirming the absence of choroidal detachment or hemorrhage and posterior segment mass lesion, using B-scan ultrasonography or ophthalmoscopy [6, 9, 15].

All the 9 patients underwent phaco-IOL-GSL with a wide range of peripheral anterior synechiae (PAS, over 180 degrees) (Table 1). Phaco-IOL-GSL was performed by Dr. Weihua Pan as previously described [4]. Three kinds of IOLs, including AcrySof SA60AT (Alcon Surgical Inc, Fort Worth, Texas, USA), Tecnis ZCB00 (Abbott Medical Optics Inc, Santa Ana, California, USA) and Softec HD (Lenstec Inc., St. Petersburg, FL, USA) were implanted during surgery. Antiglaucoma medication and cycloplegics, aqueous suppressants, and hyperosmotics agents were primarily used to treat all the patients. VHZI, either via anterior chamber or pars plana approach, would be adopted in cases the medications failed to deepen anterior chamber with or without IOP reduction within three days. VHZI via anterior chamber approach was solely performed by Dr. Weihua Pan, while VHZI via pars plana approach was corporately performed by Dr. Pan and Dr. Bin Zheng. The first 5 patients underwent VHZI via anterior chamber approach, and the last 4 patients underwent VHZI via pars plana approach. The success of surgery was defined by deepening of the anterior chamber centrally with no peripheral iridocorneal touch. This study conformed to the tenets of Declaration of Helsinki.

### 2.2. Preoperative Examination

Past ocular history, including the initial diagnosis and hyperopia (axial length of less than 22 mm) before phaco-IOL-GSL surgery, patient demographics, and the type of IOLs inserted were recorded (Table 1). Preoperative and postoperative IOP, best corrected visual acuity (BCVA), and the number of antiglaucoma medications were also recorded.

### 2.3. Surgical Technique

#### 2.3.1. VHZI via Anterior Chamber Approach

VHZI was done similarly to a previous study [10]. First, a 23-G incision was made at the peripheral inferior temporal sector of the cornea and connected with an anterior chamber infusion cannula. Another corneal incision was made at the superior temporal sector or superior nasal sector, and Viscoat® (Bausch and Lomb, Shandong, China) was infused into the anterior chamber. Iridotomy was performed through the incision using a Wescott scissor and a toothed forcep. Hyaloidectomy and zonulectomy were performed through the peripheral iris defect to set up a pathway between the anterior chamber and the vitreous cavity. We repeatedly carried out vitrectomy around the pathway to reduce the risk of postoperative reobstruction of the tunnel created.

#### 2.3.2. VHZI via Pars Plana Approach

VHZI procedure was carried out via a posterior segment approach. Anterior vitrectomy was performed with a standard three-port technique. Three 23-G pars plana incisions were made at the inferior temporal sector, superior temporal sector, and superior nasal sector. An infusion cannula was placed at the incision of the inferior temporal sector, two 23-G pars plana instruments were inserted through the superior quadrants, and the anterior vitrectomy was performed with 360° vitreous shaving, but without posterior vitreous body removal. Subsequently, hyaloidectomy, zonulectomy, and peripheral iridectomy were performed. The vitreous cutter was then placed facing up from the vitreous chamber. We then created a tunnel between the anterior and vitreous chamber. Repeated vitrectomy around the hyaloido-zonulectomy and peripheral iridectomy sites, immediately after anterior vitrectomy was performed to reduce the risk of postoperative tunnel obstruction. We confirmed successful tunnel creation by observing the outflow of the perfusion from the corneal incision attained by compressing corneal regions away from the incision.

Two corneal incisions were made using the original channel of the cataract surgery, followed by Viscoat® infusion into the anterior chamber. Visco-GSL and mechanical GSL were performed using a 23G ophthalmic endoscope (OE) probe (URAM E2, USA ENDO OPTIKS) to release PAS until all 360 degrees trabecular meshwork was observed in patients with PAS for both procedures.

Postoperatively, all patients were prescribed topical use of tobramycin and dexamethasone eye cream for 1 week (1×/d), tobramycin and dexamethasone eyedrops for 4 weeks (4×/d), and nonsteroidal anti-inflammatory eyedrops for 4 weeks (4×/d).

### 2.4. Statistical Analysis

Descriptive and inferential statistics were performed using Statistical Package for Social Studies (SPSS 24; IBM Corp, Armonk, NY, USA). Normality of the variables was tested using the Shapiro–Wilk test. Continuous normally distributed data were represented by mean and standard deviation. Datasets not normally distributed were represented by median and the first and third quartiles. A paired *t*-test was used to compare the IOP at presentation and at the final follow-up. The Wilcoxon signed-rank test was used to compare the logarithm of the minimum angle of resolution (logMAR) BCVA and the number of antiglaucoma medications at presentation and at the final follow-up. A Fisher's exact test was used to compare the recurrence rate between the groups of patients underwent VHZI via anterior chamber approach and pars plana approach. A *P* value of <0.05 was considered statistically significant.

## 3. Results

We treated 652 eyes (475 patients) with phaco-IOL-GSL in the Affiliated Eye Hospital of Wenzhou Medical University from May 2015 to February 2018. Fluid misdirection syndrome was diagnosed in 9 patients (1.4%). These patients had an average age of 60.8 ± 10.4 (range, 50–82) years. The male to female patient ratio was 1 : 8.

The medical history of the 9 patients is summarized in Table 1. Patient 1 had acute glaucoma, while the other 8 cases demonstrated chronic angle closure glaucoma (CACG). Patient 7 showed a weakness in the lens zonules. All patients had previously undergone identical surgical procedures of phaco-IOL-GSL. The interval between phaco-IOL-GSL and fluid misdirection syndrome was 11 ± 13.9(range, 4–47) days. The mean axial length was 21.35 ± 0.84 (range, 20–22.43) mm. The median diagnosis to surgery interval time was 6 days (first and third quartiles, 4.5 and 11.0 days). The median follow-up period was 8 months (first and third quartiles, 6 and 13 months, respectively).

**Figure 1:**
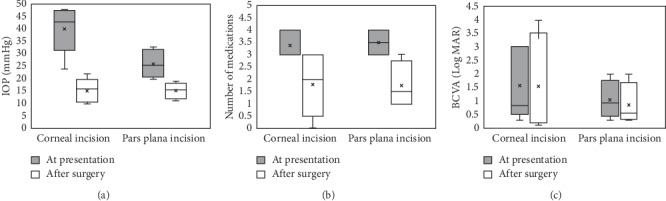
The preoperative and postoperative (a) IOP, (b) number of medications, and (c) BCVA of patients underwent VHZI via corneal incision or pars plana incision.

For patients who underwent VHZI via anterior chamber approach, success was achieved in all 5 cases. We observed a significant decline in the mean IOP values from 40.2 ± 9.7 mm Hg at presentation to 15.2 ± 4.8 mm Hg after surgery (*P*=0.01) (Figure 1(a)). Although we saw a decrease in the median of the number of antiglaucoma medications from 3 (first and third quartiles, 3 and 4) at presentation to 2 (first and third quartiles, 0.5 and 3) after surgery, the reduction was not statistically significant (*P*=0.066) (Figure 1(b)). The median BCVA in logMAR was 0.82 (first and third quartiles, 0.50 and 3.0) at presentation and was 0.3 (first and third quartiles, 0.2 and 3.5) after surgery. The difference was not statistically significant (*P*=0.071) (Figure 1(c)). A 58% (range, 33%–78%) decline in IOP for patients who underwent VHZI via anterior chamber approach and a 45% (range, 5%–75%) decline in IOP for patients who underwent VHZI via pars plana approach at the last follow-up. We observed relapse of FMS in 2 of the 5 cases (40%). Patient 4 showed recurrence in 9 days postoperatively, demonstrating shallowing of the anterior chamber with the IOP increasing to 28 mm Hg. Patient 5 showed recurrence in 3 days postoperatively, with a shallowed anterior chamber and the IOP increased to 19 mm Hg. A layer of membrane, which might be fibrin exudates, was observed covering the peripheral iridectomy in both cases. The anterior chamber deepened immediately after membrane lysis using Nd:YAG laser through peripheral iridectomy in both cases.

For patients who underwent VHZI via pars plana approach, success was achieved in all 4 cases. Mean IOP reduced from 26.0 ± 5.7 mm Hg at presentation to 15.2 ± 3.3 mm Hg after surgery; however, the difference was not statistically significant (*P*=0.092) (Figure 1(a)). The median of the number of antiglaucoma medications reduced from 3.5 (first and third quartiles, 3 and 4) at presentation to 1.5 (first and third quartiles, 1.0 and 2.75) after surgery, which also was not statistically significant (*P*=0.059) (Figure 1(b)). The median BCVA in logMAR was 0.91 (first and third quartiles, 0.43 and 1.75) at presentation and was 0.55 (first and third quartiles, 0.32 and 1.67) after surgery. Also, the difference was not statistically significant (*P*=0.18) (Figure 1(c)). No detectable relapse was found in any of the 4 cases, however, no statistically significant difference was found between the two groups with the small sample size (*P*=0.444).

## 4. Discussion

Fluid misdirection syndrome, as first described by Von Graefe in 1869, is characterized by elevated IOP and a very shallow anterior chamber with the absence of pupillary block, choroidal effusion or hemorrhage and no noticeable pathology of the iris-lens diaphragm [8, 16]. FMS secondary to a different glaucoma treatment is extremely rare, with the incidence rate from 0.03% to 6% [7, 17, 18]. The exact pathophysiology of fluid misdirection syndrome is still unclear. Currently, FMS occurrence is assumed to be primarily related to the presence of an abnormal anatomic relationship between the ciliary processes, the crystalline lens, or IOL and anterior vitreous face, leading to the misdirection of aqueous fluid into the vitreous cavity [8, 10, 16, 18–20].

Previously, VHZI has been considered effective in re-establishing the aqueous flow between the anterior chamber and vitreous chamber [7, 9–12, 14], which could be achieved through anterior chamber or pars plana approach. However, most studies only reported the results of VHZI using one of the two methods. Here, we try to compare the outcomes of those two approaches, which might help us to plan a more effective surgery for FMS treatment.

The initial VHZI was successful in most cases. Here, the visual acuity of patient 1 decreased from hand movement from 30 cm away preoperatively to light perception postoperatively. In this case, the preoperative IOP was high (43 mm Hg), and the duration of diagnosis to surgery interval was long (15 days), consistent with a previous study [15]. The IOP was reduced after treatment in all cases except patient 4, who showed an IOP of 22 mm Hg despite using 3 kinds of antiglaucoma medications. We postulated that it might be due to the recurrence of PAS and the impairment of trabecular meshwork function.

**Figure 2:**
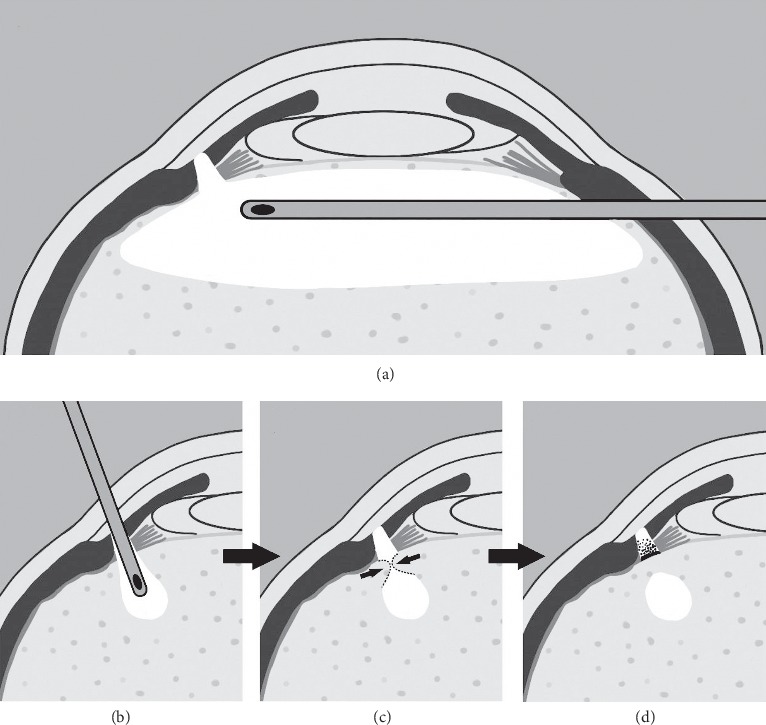
The schematic showing the anterior vitrectomy via (a) pars plana approach or (b–d) anterior chamber approach. (a) The vitrector inserted through the pars plana incision, approximately parallel to the plane of the iris. As a result, there is enough space for the movement of the vitrector, which is helpful to adequately create a wide disruption of the anterior hyaloid face and remove the anterior vitreous body around the tunnel. (b) The vitrector inserted through the peripheral iris defect into the vitreous cavity vertically. For the space of the vitrector, movement is limited to the vicinity of the incision, and the operative area cannot be observed during anterior vitrectomy, and the anterior vitreous around the passage is hard to be removed. (c) The residual vitreous around the passage after VHZI via anterior chamber approach may invade and block the passage. (d) The deposition of the fibrin exudates may form a membrane on the front surface of the residual vitreous around the channel.

FMS recurrence after VHZI has previously been well documented. FMS relapse rate after VHZI via pars plana incision varies significantly in different studies (0% by Sharma et al. [11], Bitrian et al. [9, 12], 10.1% by Al Bin Ali et al. [15], and 66% by Debrouwere et al. [6]). Similarly, FMS relapse rate after VHZI via anterior chamber approach also varies significantly (0% by Lois et al. [10], Zarnowski et al. [21, 22], and 40% by Madgula and Anand [14]). However, previous studies have failed to conclude if there is a difference in relapse rates when comparing the two surgical approaches. Our results showed that the relapse rate was significantly different between the two groups, at 40% and 0% with VHZI via anterior chamber and pars plana approach, respectively. The rationale behind such a difference between the two groups in our study was not yet fully understood. During this surgery the anterior vitreous around the tunnel needs to be adequately removed. When through pars plana approach, the vitrector can be manipulated with a higher degree of freedom, and an auxiliary lens can clarify and magnify the surgical visual field. Additionally, the vitreous cutter is facing upward when hyaloidectomy, zonulectomy, and peripheral iridectomy are performed, which help to remove the vitreous body near anterior hyaloid face as thoroughly as possible. Therefore, the anterior vitreous around the tunnel could be easy to remove adequately (Figure 2(a)). In comparison, it is difficult to remove the anterior vitreous around the tunnel completely via the anterior chamber approach. In fact, very limited anterior vitrectomy was performed in the latter approach. First, the vitrector was inserted through the peripheral iris directing into the vitreous cavity vertically, so its manipulation is limited to the vicinity of the incision. Second, as the incision is vertical, the operative area cannot be observed during anterior vitrectomy. Furthermore, extra attention should be paid to avoid damaging the peripheral retina and ciliary body (Figure 2(b)). In our case series, 8 of 9 eyes were followed up for more than 6 months, and membrane covering peripheral iridectomy was observed in 2 cases with recurrence of FMS. We considered that the membrane might be formed by deposition of fibrin exudates on the front surface of the residual vitreous around the channel (Figures 2(c) and 2(d)), which was reported in the previous study [14].

**Figure 3:**
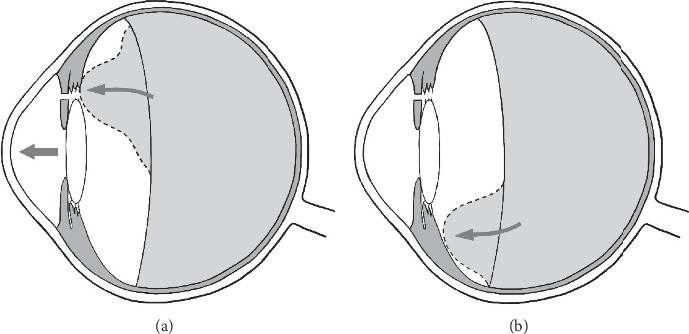
The schematic showed (a) the hypothesis of the remnant vitreous moving forward and blocking the created passage after incomplete removal of the vitreous (comprehensive vitrectomy). (b) Residual posterior vitreous body does not easily block the upper tunnel directly for its more likely to accumulate at the lower part of vitreous cavity due to gravity.

Debrouwere et al. considered that complete pars plana vitrectomy combined iridectomy and zonulectomy was more effective than anterior vitrectomy combined iridectomy and zonulectomy in resolution of FMS in their retrospective case series. They inferred that presumably because the remaining posterior vitreous obstruct the created tunnel in the patients who underwent anterior vitrectomy (Figure 3(a)). However, comprehensive anterior vitrectomy via pars plana approach combined with iridectomy and zonulectomy has shown to be an effective strategy for FMS in other studies [9, 11, 12, 15]. To our knowledge, the flow tunnel created between anterior chamber and posterior chamber should always be built at the upper quadrant. Residual posterior vitreous body is more likely to accumulate at the lower part of vitreous cavity due to gravity, which does not easily block the upper tunnel directly (Figure 3(b)). Our study showed that the VHZI via pars plana approach can achieve effective results, which is consistent with the previous studies [9, 11, 12, 15].

The two cases of relapse FMS in our study were both solved by Nd:YAG laser fibrinolysis. However, only one of the four relapse cases was successfully treated with the Nd:YAG laser fibrinolysis and anterior hyaloidectomy in the study of Madgula and Anand. The others were resolved through repeated anterior vitrectomy and cyclodiode laser [14]. We hypothesize that Nd:YAG laser may be ineffective if the tunnel between anterior and posterior chambers is significantly blocked by residual vitreous. Repeated anterior vitrectomy helps to remove residual vitreous around the tunnel and re-establish the tunnel between the anterior chamber and vitreous cavity, which may be an alternative option in recurrent FMS unresponsive to Nd:YAG laser therapy.

Although VHZI via pars plana approach has three scleral incisions, while VHZI via anterior chamber approach has only two, previous studies did not show any significant difference in the comparative analysis of the rate of postoperative complications between the two methods [9, 12–14, 21]. Recently, a retrospective study with a larger sample size showed that a very low postoperative complications of retinal detachment (2 eyes, 3%) and endophthalmitis (1 eye, 1%) after VHZI via pars plana approach [15], justifying the safety of this approach. Here, in this study, we did not observe any complications during or after VHZI in any of the two groups.

The main limitation of our study is that it was a retrospective study with small sample size. A prospective, randomized study comparing the outcomes of different approaches of VHZI would be necessary to reach a more definite conclusion. However, owing to the low incidence of FMS and the long duration required to obtain enough cases (1.4%, 9/652 eyes, 3 years, as in our center), a prospective study with appropriate sample size with long-term outcomes will take a significantly longer time to accomplish.

## 5. Conclusion

In summary, FMS is a rare, but severe potential complication observed postoperatively in phaco-IOL-GSL surgical procedure. Compared to VHZI via anterior chamber approach, VHZI via pars plana approach might be a more effective procedure in treating FMS. The anterior vitreous can be removed more completely with VHZI via pars plana approach, which may therefore reduce the probability of postsurgical reblocking of the tunnel between anterior chamber and vitreous cavity.

## Figures and Tables

**Table 1:** Preoperative data and results of VHZI of the nine patients.

Patient number	Eye	History	IOLs inserted during phaco-IOL-GSL1	PAS2 before phaco-IOL-GSL (degrees)	Incision site of VHZI3	Axial length (mm)	Diagnosis to surgery interval (days)	Follow-up (months)	Relapse
1	LE	Acute glaucoma	SOFTEC HD	360	Cornea	22.30	15	6	No
2	LE	cacg^4^	SOFTEC HD	270	Cornea	21.58	7	31	No
3	RE	cacg	SOFTEC HD	360	Cornea	21.39	47	7	No
4	LE	cacg	AcrySof SA60AT	270	Cornea	21.32	6	6	Yes^#^
5	RE	cacg	AcrySof SA60AT	270	Cornea	21.41	5	13	Yes^##^
6	LE	cacg, after LPI^5^	Tecnis ZCB00	360	Pars plana	20.05	4	3	No
7	RE	cacg, weakness of lens zonules	SOFTEC HD	360	Pars plana	22.43	5	9	No
8	LE	cacg	Tecnis ZCB00	270	Pars plana	21.72	4	13	No
9	LE	cacg	AcrySof SA60AT	270	Pars plana	20.00	6	8	No

LE: left eye; RE: right eye, 1: phaco-IOL-GSL—phacoemulsification with intraocular lens implantation combined with goniosynechialysis, 2: PAS—peripheral anterior synechiae, 3: VHZI—anterior vitrectomy, hyaloidectomy, zonulectomy, and peripheral iridectomy. 4: cacg—chronic angle closure glaucoma. 5: LPI—laser peripheral iridotomy.^#^Relapse 9 days after VHZI.^##^Relapse 3 days after VHZI.

## Data Availability

The datasets generated and analyzed during the current study are available from the corresponding author on reasonable request.
